# Post-Resettlement Food Insecurity: Afghan Refugees and Challenges of the New Environment

**DOI:** 10.3390/ijerph20105846

**Published:** 2023-05-17

**Authors:** Zahra Goliaei, Mariaelena Gonzalez, Karina Diaz Rios, Mamata Pokhrel, Nancy J. Burke

**Affiliations:** 1 Public Health Program, College of Education and Health Science, Touro University of California, Vallejo, CA 94592, USA; 2 Department of Public Health, School of Social Science, Humanities, & Arts, The University of California Merced, Merced, CA 95343, USA

**Keywords:** food access, food insecurity, Afghan refugees, food assistance, resettlement, United States

## Abstract

Background: Lack of access to adequate, safe, and nutritious food is a major concern for the Afghan population due to ongoing war and humanitarian crises. Recently resettled Afghan refugees in the US continue to face challenges securing adequate, nutritious food resources in new environments. This study examined Afghan refugees’ food access and insecurity in the San Joaquin Valley, California. Methods: Semi-structured, in-depth interviews were conducted to collect the perspectives and experiences of key informants and newly arrived Afghan refugees. Results: This study highlights environmental and structural factors (availability and accessibility of grocery stores; availability of religious-appropriate items in the stores; the public benefit received by a family; and public transportation) and individual factors (religious and cultural practices; financial and language barriers) as major determinants of post-resettlement food insecurity. Conclusion: Increasing the accessibility and affordability of culturally and religiously appropriate food items within the US food system, enhancing the collaboration of community volunteers and resettlement organizations in the direct assistance of new families, and providing continuous access to public benefits are possible steps to mitigate the risk of food insecurity among Afghan refugees. This study suggests a continuous examination of the degree of food insecurity in this population and its attendant health impacts.

## 1. Introduction

As the humanitarian crisis increased in Afghanistan, the United States evacuated more than 74,000 Afghans and facilitated their resettlement in different locations across the states (fiscal year 2021) [1]. To support the acceptance of refugees, on 8 September 2022, the US President announced a cap of 125,000 new refugees for the 2023 fiscal year [2]. Refugees generally leave their countries due to insecure environments [3], which leads to encountering multiple challenges before and during the migration process and after resettlement that can negatively impact refugees’ health outcomes [4,5]. Among those challenges, food insecurity has been a major ongoing threat to refugees’ health [6,7].

Food insecurity is defined as “a household-level economic and social condition of limited or uncertain access to adequate food for an active, healthy life that may or may not lead to periodic reductions in food” [8] and is associated with poor health outcomes such as anemia, malnutrition (including obesity), diabetes, and hypertension [9]. According to the United Nations High Commissioner for Refugees (UNHCR)’s most recent report, around 82 percent of internally displaced people (IDPs) and 67 percent of refugees and asylum seekers originate from countries that experienced severe food crises [10]. The prevalence of food insecurity has been exacerbated during the COVID-19 pandemic for the general population and for those resettled in the US [11,12].

Although studies have identified poverty as a determinant of food insecurity for the general US population [13], refugees living in the US face added challenges [14]. Lack of familiarity with the retail food environment and its products, as well as language barriers, were reported to be linked with food access difficulties and the risk of further food insecurity among refugees [14,15,16]. Difficulty accessing needed ingredients, lack of interpersonal relationships in new neighborhoods, unique cooking customs, and acculturation to the host countries’ eating patterns may create changes in families’ dietary patterns [17]. These changes in dietary patterns post-resettlement have been found to significantly contribute to refugees’ health deterioration [17]. Few studies have focused on the interaction between environmental factors and individual factors that shape refugees’ food choices, nutritional status, and risk of food insecurity [15,16].

Food insecurity is a global concern for Afghan refugees; as the humanitarian crisis in Afghanistan intensifies, millions of Afghans experience severe food shortages [18]. Hunger statistics data (percentage of the population whose food intake is insufficient to meet dietary energy requirements continuously) from Afghanistan reports an increase of six percent, from 23% in 2017 to 29.8% in 2020 [19]. A recent report from Afghanistan indicated that in 2021, nearly 19 million Afghans would experience acute food insecurity [20]. The challenges of food insecurity are exacerbated upon arrival in countries like the United States. The majority of the Afghan population (99.7% in the existing resources) is reported to be followers of Islam [21], which could have an impact on most aspects of their lives, including their food choices. For many Afghan refugees, as for other Muslims, food is preferentially restricted to *halal* foods—those that adhere to Islamic law. *Halal* meat requires a specific method of slaughter [22]. *Halal* also indicates that the product is free of any pork fragments, parts of dead animals, blood, or intoxicants such as alcohol [22]. The global *halal* food market demand experienced a rapid increase of about 8.8% between 2017 and 2021 [23]. In the US, the number of places that offer *halal* meat items increased from 200 in 1998 to more than 7500 in 2016, which is still considered a small number compared to the whole US food market [24]. Based on the most recent Google search, there is only one *halal* butcher store and four *halal* markets in the study area [25].

Despite the growing body of research on the impact of the physical environment (neighborhood geographic, economic, and social characteristics) on food insecurity in different communities in the US [26,27,28], little research has examined the impact of neighborhoods and food access on the food insecurity of Afghan refugees in the United States. Little is known about how Afghan refugees adapt to their new food environment, address their religion-specific food needs, and how that could exacerbate the Afghan family’s risk of post-resettlement food insecurity. This study employed a qualitative approach to explore Afghan refugees’ encounters, experiences, and challenges in the new food environment to better understand the determinants and root causes of food insecurity in the newly arrived families resettled in California’s San Joaquin Valley, where in the most recent report of 2022, 77% of the population reported below the federal nutrition program threshold and 12.1% below the overall rate of food insecurity [29].

## 2. Methods

This study analyzed the existing data collected in 2018–2019 as part of a study aimed at exploring the perspectives and experiences of recently arrived Afghan refugees regarding food access and food environment navigation in the San Joaquin Valley, California. Data was collected through in-depth, semi-structured interviews with Afghan refugee families and key informants in three major cities in the area. We used purposeful sampling to recruit key informants from service providers working for organizations (governmental or non-governmental) directly serving recently arrived Afghan refugees, religious and community leaders, and community volunteers who directly support newly arrived refugees. Refugee participants were identified and contacted through snowball sampling. Key informants who participated in the study introduced the study to the Afghan families with whom they were in contact, and those families who agreed to participate in the study were asked to introduce the study to other families. After initial recruiting, participants received informed consent via email in advance. The informed consent was also read to the participants by the interviewer at the beginning of each interview. Oral consent was obtained and audio-recorded at the onset of each interview. None of the study participants disagreed with the informed consent, and no participant disagreed with being audio recorded. If the interview was conducted at a family’s house, the wife and husband participated jointly. All the interviews were conducted by the first author, who is proficient in Farsi and English and trained in conducting qualitative interviews. Key informants’ interviews were conducted in person and in English. Refugee interviews were conducted in either Farsi or English, depending on the participants’ choice. All the refugee participants in this study were comfortable understanding the interviewer’s Faris dialectic, and there were no Pashto-speaking participants in this study.

A preliminary semi-structured interview guide was developed based on the literature reviews and the research team’s experience working with refugees in different areas. The interview guide for key informants included questions about their role in refugee resettlement; their perspectives on refugees’ food choices, shopping processes, financial resources for grocery shopping, the adequacy of available food items, and the availability of local food services; and other barriers related to food access and possible solutions. The interview guide developed for Afghan refugee families included questions on individuals’ or families’ access to needed grocery items after arrival, experience with food shopping in the United States, navigation of the physical environment, barriers to obtaining needed items, food preparation, and eating habits after resettlement. At the end of the interviews, the interviewer (the first author) summarized the participants’ responses and allowed them to change their responses or provide clarification. Each interview lasted approximately one hour. After the study, all participants received an e-gift card as a token of appreciation for their time and support of the study. All interviews were audio-recorded and transcribed verbatim. English interviews were transcribed by GMR Transcription [30], a professional transcription service founded in 2004, and 99% accuracy was guaranteed. Moreover, Farsi interviews were transcribed by the first author, a native Farsi speaker. The University of California, Merced Institutional Review Board approved the study.

Data analysis and interpretation included concomitant consultation with the available literature, field notes, and analytical memos. We utilized a thematic analysis approach [31]. After each interview was completed, we conducted line-by-line coding using ATLAS.ti (Windows version 19.0.8.0) to support the coding process [32]. Interviews with key informants and refugees were coded with the same coding system. Emerging codes were used to create prompts for necessary changes in interview guides. Interviews were stopped when there were no emerging codes in the two final interviews. The next step in data analysis was to develop descriptive themes around major concepts discussed from key informants’ perspectives and refugee families’ experiences with food access and its determinants in the post-resettlement environment for Afghan refugees in San Joaquin Valley, California.

## 3. Results

Twenty-four interviews were conducted overall. We completed twelve interviews with key informants and twelve interviews with Afghan families resettled in the area for less than two years. No demographic information was collected from participants. No detailed demographic information was collected from the participants. Based on the recruiting charts, refugee interviews included one woman and three men, and the rest, both wives and husbands, participated jointly. All participants were 18–45 years old and had dependents in their household (varying between the number of children and, in some cases, including grandparents). For the key informant interviews, we had five women and seven men. Three interviewees were religious or community leaders; three were community volunteers; and the rest were staff of different governmental and non-governmental organizations serving refugees in the study area.

The major themes emerging from our study participants’ perspectives and experiences on major determinants of post-resettlement food insecurity in Afghan refugee communities were lack of familiarity with the new environment (navigation of grocery stores), religious and cultural practices (excessive barriers and restrictions), transportation and housing (access limitation), cost of grocery items (lack of financial literacy), and limitations of food banks and food pantries (lack of healthy options).

### 3.1. Lack of Familiarity with the New Environment

Navigation in Grocery Accessing and navigating grocery stores for newly arrived refugees is challenging and fear-inducing. When a family resettles in a new place, it takes time to become familiar with the physical environment, including the streets around their home, grocery stores nearby, and ways to access neighborhood resources. A refugee male participant mentioned, *“When we came here, I did not know anything. Where to go? Where to shop? What are the foods? We started going out with my kids and finding places. Sometimes we got lost”*. Another male participant added: *“It would take about an hour to find and reach a store and shop for something, and then an hour again to come back. It was about two hours, and when we came back, our wives were so worried. Our wives were very afraid because we did not know anything. We did not know where to go. Thus, not knowing anything made everyone very afraid every time we were out shopping”*.

Some families received support from family and friends who had resettled in the area earlier or from community volunteers. As one of the refugee participants reported, *“When we arrived first, the Afghan people who came before us helped us find the stores with Afghan food”.* Community members usually help newly arrived families find stores in the area and access necessary items. A female key informant, a community volunteer, told us, *“They navigate the stores through networking; when they come first, the religious center is their GPS, I would say”.* Another female key informant, staff of one of the local resettlement centers, also noted the value of this community support: *“Nowadays, the community takes good care of newly arrived families. There are members of the Afghan community who will typically come alongside them and show them the ropes, show them the places to buy the best things, where to buy the least expensive things, and how to have access to them”.* Families without this community support had to explore the area on their own to find and access the needed stores. One of the refugee women mentioned: *“No one showed me the neighborhood stores. I was going out on my own and walking around and finding stores. Slowly, I learned about the stores and what they had”.* A female community volunteer also emphasized the hardship refugees face when navigating on their own and noted: *“Without guidance, finding the food and the place of Afghan food they need is very difficult. Doing it alone is difficult. They should have a guide. Someone should guide them so they can shop”.*

In addition to the challenges of unfamiliar neighborhoods and geographic locations, a lack of familiarity with grocery stores’ organization and payment systems was also reported as intimidating and creating barriers to accessing required food items for recent arrival refugees. As one refugee told us, *“The problem was we did not know where to find what; we had to go to a store multiple times to figure out where to find what. After we went sometimes, we learned about those stores and knew where the items were located”.* Another refugee man compared his experience from back home with the new environment: *“From the place, we came to these stores; these systems were not there. We were very nervous. How should we pay? How does the system work, and what should we buy? After a month, it became easier. The first month was very hard when we went to those stores and saw their systems”.* Some resettlement agencies offered a short training for families after their arrival, which included a tour of the local neighborhood, assistance with public transportation navigation, and details about nearby grocery stores. However, not all agencies offer such tours, and this tour is available to a limited number of families.

Along with difficulty navigating the stores, newly arrived families reported difficulties identifying available pre-packaged items and most needed help deciding what they could shop for or were looking for. One refugee family told us: *“The first week was hard; it was hard because we did not know the food.”* One of the female staff members of the resettlement center’ also noted: *“I think in the beginning, it is overwhelming because it is a big, crazy grocery store. I think they just kind of shop around.”* The training provided by resettlement agencies, while useful, usually does not include any introduction to the items available in the grocery store, and a lack of familiarity with US products occasionally leads to buying the wrong products. Another refugee family disclosed, *“There were times that we bought an ingredient and brought it home to cook with, and in-home, we realized it was not what we wanted, or the one we bought, we cannot use it”.*

Navigating an unfamiliar environment could also be more challenging for families who experience language barriers. Some of the key informants in this study mentioned that, most recently, resettled Afghan men are mostly well-educated and have English language skills. However, single mothers and elderly individuals struggle to communicate in English and have more difficulty shopping for food items in US grocery stores. As one of the community volunteers told us, *“For some families*, *when they first arrived, we almost had to use the picture-shopping list because there was a translation difficulty and they did not know the items. We cannot communicate; we just use pictures, or sometimes we just bring grocery items with us and ask them to circle items they are looking for so we can locate them at the stores”*.

### 3.2. Religious and Cultural Practices: Excessive Barriers

As most Afghan families practice Islam, immediately after arrival, they look for appropriate religious items, such as *halal* meat. The number of markets that offer *halal* items in this area is limited, and they often only offer limited items that are mainly frozen. One community volunteer observed: *“Accessing halal meat is difficult, and there is only one halal butcher in the area. The butcher store is usually far from where most families resettled, and families usually need rides or travel long distances via public transportation to shop for fresh meat”*. One of our refugee participants also shared: *“For meat, we had to take one bus to the central terminal and the second bus to another city”*. Along with the hardship of accessing *halal* items, the price of *halal* meat is much higher than other meat products available in general grocery stores. A resettlement agency staff member reported: *“The meat is hard to come by here because most of them are still trying to observe halal meat, which is unavailable in our communities”*. A community volunteer (female) also noted, *“The price is different. For meat, it is up to $2 extra. The chicken here in stores is not halal, and it is 1.18–1.19 (dollars) per pound, but the halal chicken is about 2.50–2.60 per pound”*. Due to the higher prices, families who only consume *halal* meat (as opposed to those who consume non-*halal* US products) may either eat less meat during the month or spend a higher proportion of their budget to purchase needed protein items. A religious leader (male) described the effects of these different prices: *“You know that the regular processed chicken in the markets is much cheaper than the halal chicken. If families are limited on cash, if they are going to buy, for example, two pounds of halal chicken, they can get it for the same price, about four or five pounds, from outside. Especially looking at the family size. I would say the average size of a family is five to six individuals. Thus, imagine if they are going to spend this money only on buying meat; where is the money that is going to be spent on other groceries as well?”* The higher meat price can cause families to run out of food assistance benefits more quickly compared to other families. A refugee participant told us, *“Sometimes, the kids are craving meat, so we have to pay more and buy halal meat, and again, we are running out of food stamps”*.

Another emerging concern in this study was unfamiliarity with the ingredients and with pre-packaged items available in US stores and fear of whether those items are religiously or culturally appropriate, which may limit the family’s choice and willingness to try those products. A community volunteer female participant remarked, *“Getting safe food items is not something that is easy, especially canned food. I got some canned garbanzo beans and red beans for refugee families. They don not know what is added to them, so I read the label to them”*. Families often prefer to shop at ethnic stores that have a familiar environment and limited items at higher prices. The ethnic stores carry religious or culturally preferred items that families may easily recognize and are familiar with the packaging and taste of. Even though the items have higher prices than similar items in US grocery stores, families prefer to shop there because they are identical to the brands and packaging, they used previously. The same volunteer also disclosed, *“They see what they would find in Afghanistan for produce and stuff, and they just buy that”.* One of the religious leaders (a male participant) also added: *“We have an Indian market; it is very small, but they go there very often and buy rice or oil they are familiar with. Thus, they spend lots of money in one minimart, which is very expensive, but they recognize the packaging more there, and they recognize the products more, so they are comfortable there.”*

We also realized that service providers and community volunteers might also have difficulty navigating *halal* items and be confused by refugee families’ different practices. Some key informants in our study mentioned that refugee families used *halal* to refer to all culturally preferred items, which makes it confusing for providers who support refugee families. A community volunteer admitted, *“I am not even familiar with all halal products. Some families even look for halal flour, halal oil, and obviously halal meat”.*

### 3.3. Transportation and Housing: Access Limitations

In addition to navigating grocery stores in the new environment, newly arrived families must navigate public transportation and learn how to best access the stores based on their housing locations. As a result, neighborhoods’ structural resources could highly shape families’ access and food choices. A male community volunteer disclosed, *“Some families live far away, and transportation, of course, is a major problem for them to come back and forth to the grocery stores”*. Most recently arrived refugee families have not had access to personal transportation for several months and would highly rely on public transportation or shop at local stores within walking distance of their homes. Local stores tend to have fewer items, which are higher priced and lower quality, than larger grocery stores. A female community volunteer reported, *“The part of town that the majority of families are living in does not have grocery stores within walking distance. They would need to walk almost two miles to get to a regular grocery store instead of a local market like a gas station so that they could buy something. There are some neighborhood markets, but they are very expensive”*.

Recent arrivals also must rely on volunteers or other community members for transportation to the grocery stores and ethnic markets, which could limit the shopping locations and timing. One resettlement agency staff member cited: “A family who does not have a car after a *year is still dependent on the volunteers and their help for rides to grocery stores. A family that purchases a car is more independent, and they go more to grocery stores on their own, so in one year, it depends if they can get personal transportation”.* Using public transportation could be confusing and time-consuming and varies based on the city of resettlement; however, after some time, most families learn how to use public transportation and highly utilize it, even though it would take a long time to commute to the store with public transportation. This could also bring up the issue of food safety and the question of which items could be transported safely during these long trips. One of the religious leaders explained: *“Once they know where to go, most families use buses. Resettlement agencies give the families vouchers to use the buses. Once they know the bus route, they can go on their own. Usually, the trip that would take about 30 min takes them two hours to reach because of the different bus stations where they need to stop and all that”*, As it is difficult to schedule a ride to the stores, families may prefer to shop in bulk to avoid running out quickly. However, shopping in bulk could lead to spending a significant portion of the food assistance benefits on nonperishable items, possibly running out of food assistance earlier in the month. Running out of food assistance could result in being unable to access fresh ingredients, including fresh produce, eggs, milk, vegetables, and fruits, closer to the end of the month or for a long period of time. One female community volunteer observed: *“At the beginning of the month, they will choose to buy 150 pounds of flour and five packs of oil, and then they run out of money every month”*.

### 3.4. Food Banks and Food Pantries: Limited Utilization and Options

Some key informants in this study observed that, unlike most low-income families in the US, refugee families may not be willing to use food bank items in areas where food banks and pantries are available. Most food pantry items are not familiar to families or may not be culturally appropriate for ethnic or religious minorities. One female community volunteer cited, *“There are different food ministries and food banks that help. The families typically do not use them a lot just because it is not the food that they would typically eat. At some point, if there are things that might be helpful to them, then they will take them, but for the most part, it is not the same. It is not what they would normally eat. For example, I guess the best way to break it down would be for canned goods and different things like that. They typically will not eat the same chilis that a food bank would provide, but if there are ever times when there’s fresh produce available, which we have seen often, like fruits and vegetables, spinach, tomatoes, and different things like that, from some of the local markets that might have a surplus and would donate some, then you see very active participation in receiving those items”.*

While other programs such as the Salvation Army and Food for Hunger may be available in the area, refugee families often feel uncomfortable participating in them, especially because they require standing in line with their families; thus, like food banks, these programs are underutilized. As one of the community volunteers explained: *“There are some meal programs that offer free meals, but for these families to take their children to a location to get the free meals, they would, I think, rather just eat bread or whatever they can make on their own. They do not want to go and stand in line to get free meals with their kids. They do not want to do that*”.

The key informant participants were also concerned about the possible impact of a lack of access to fresh items later in the month and excessive access to food of low nutritional qualities (particularly sugary beverages and candy) in US grocery stores on the families’ eating habits, which can lead to potential adverse health outcomes. One of the community leaders noted: *“Families all eat the same thing; if they are younger or older, they all eat one food. The little kids eat the food of the elders; they should have something specific for them, but it is a bit expensive, and they may not be able to afford it”.* Some study participants observed weight changes in family members within a few months of their arrival. Families with children start to adapt to the choices of the US food environment in their diet as children are introduced to US food items at their schools and start using prepared and pre-packaged meals, junk food, and fast foods regularly in their regular diet. One refugee woman told us, *“Our kids, because they go to school here, eat lunch and breakfast there, so they are used to the food here and are familiar with some of the foods and want them from us”*. A community volunteer also cited: *“We see that people are gaining weight because they are eating a lot more American food over time. I’d say after a year, some of them are eating at fast food restaurants”*.

### 3.5. Price of Food Items: Lack of Financial Literacy

Like other low-income families, financial hardship could be a major determinant of food choices among refugees resettled in the San Joaquin Valley, California. Key informants in this study mentioned that economic assistance through the Supplemental Nutrition Assistance Program (SNAP, formerly known as food stamps) is the primary (and sometimes only) source of shopping for food for refugee families. Our participants believed that understanding how SNAP benefits work and how to budget for a month could challenge new families. As a resettlement agency staff member noted: *“That is a main source of their food. I mean, that is support; that is a food stamp. Sometimes, that is enough. Sometimes, that is not enough. However, they should learn and practice management. Their food stamps have to get them to the end of the month. Thus, that is the only source they are using for the food”.* One of the community leader participants pointed out: *“There is no other source. They are already short on other sources. Hence, they cannot get money from other places for food; they only have food stamps. If they run out, there is nothing; they can only reduce their food. That is the only way they can manage”*. Newly arrived refugees may need support and be taught budgeting practices to make their benefits last a month. Some of the key informants emphasized that newcomers need more support and training on financial literacy, food budgeting, and spending food stamps. One community volunteer cited: *“Maybe never before in their lives have they gotten everything in one pot at the beginning of the month. I do not understand why budgeting does not seem to be a part of their lives. I would rather see them educated on how to spend than spend all their money at the beginning of the month and not have money at the end”*. In conversations with a religious leader participant, he shared: *“I have felt like they need some budgeting help. Maybe it is because food has been insecure for them in the past. Maybe never before in their lives have they gotten everything in one pot at the beginning of the month”*.

## 4. Discussion

Food insecurity, or the lack of access to enough healthy and necessary nutrient items [8], is a major threat to the health of low-income communities. Existing studies have reported on multi-dimensional barriers that impact food access and food security in migrants and indicated that food insecurity in these communities results from elements such as individual characteristics, economic resources, and structural constraints of the physical environment [15,33]. This study examined the factors impacting food access for Afghan refugees resettled in the San Joaquin Valley, California. Our findings indicated that food access and choices of recently resettled refugees in this area are affected by other complex factors, including lack of familiarity with the US food environment, transportation and language barriers, and poor budgeting abilities, which support the existing literature [15,34,35].

We highlighted the impact of being a religious minority (Muslim) and the lack of availability or higher price of *halal* items in the post-resettlement environment. Another concern related to *halal* items was uncertainty about the definition of *halal* and its application among families and service providers. Our discussion with the study participants revealed that identifying *halal* items is challenging for most Afghan refugees, especially with pre-packaged items. As a result, Afghan refugees are not utilizing most items offered by food pantries. This hesitation is not a matter of preference but of religious obligation, which could justify why, unlike other low-income families in the US, shopping for less expensive equivalent items available in US grocery stores or using food pantries may not be a desirable solution for Afghan refugees. This study also reported that for refugees, food stamps are the only source of payment for food items, and newly arrived families lack the budgeting ability to make their food stamps last for the whole month, like what has been reported in other low-income communities [35,36].

To address these concerns, we encourage refugee-serving organizations and policymakers to invest more in understanding the refugee communities’ food access barriers and needs and increase collaboration among all service providers serving the newly arrived Afghan refugees. Strong collaboration among NGOs, religious and community center leaders, and community volunteers will create cross-agency support to design and expand relevant and effective interventions to address new arrivals’ food access challenges. Ultimately, all resettlement organizations should expand their cultural orientation training to include a tour of the new neighborhood’s food markets. Community volunteers can also provide organized help with the transportation and shopping needs of the new families. Religious centers can provide educational guidelines on *halal* items available in the local area for new families and service-providing organizations’ staff, including food banks.

## 5. Limitations

This study focused on the experiences of refugees and key informants who closely work with refugees from a specific ethnic/linguistic background. Refugees who participated in this study all recently arrived and were Dari speakers. Refugees who are mainly Pashto-speaking may have additional barriers that we did not have a chance to examine. Furthermore, refugee families’ food access challenges and risk of food insecurity could vary with the length of their stay in the US. Another limitation to consider is that the resettlement area of this study is geographically distinct, with the native community being predominantly Latino, which could result in different resources being available compared to other resettlement locations. We also should consider that Afghan refugees mostly come from areas of severe hardship, like refugee camps, conflicts, and war zones. Experiences of striving for food in the past may lead families to adjust to what is currently available and underreport difficulties or a lack of access; and more likely to demonstrate appreciation and satisfaction with whatever resources they have.

There is a need for future public health research to explore the food status and risk of food insecurity among refugees with different ethnic backgrounds and among those who have been living in the US for an extended period, have established employment, and may not be eligible for federal support and food assistance anymore. The issue of changing the diets of school-aged children and the risk of further adverse health outcomes, including obesity, should also be examined in more detail in future research, along with consideration of the lack of *halal* items in school lunches. Finally, the interviews for this study were conducted before the pandemic. More studies are needed to investigate the pandemic’s impact on Afghan refugees’ food access challenges.

## 6. Conclusions

Determinants of food access for recently resettled refugees in the San Joaquin Valley, California, are complex and involve multi-dimensional individual, environmental, economic, societal, and institutional factors. While the financial situation is the major critical factor in food planning and dietary decisions for native US low-income families, for the recently arrived Afghan refugees, a combination of religious practice and unfamiliarity with the environment (physical, financial, and food systems) could enhance the impact of financial difficulties. The gap between existing resources and families’ needs could be resolved with collective effort, including increasing social support, improving the US food system, making more ethnic-appropriate food items available in US grocery stores, and directly engaging communities and resettlement agencies in supporting newcomers as they navigate the post-resettlement environment.

Although this paper focused on understanding the experiences of a small community of refugees resettled in the United States, most of the challenges discussed should be considered while serving other Muslim refugees across the US. With the increasing rate of refugees coming from conflicted areas, including Syria, East Asia, and North Africa, there is a need for a continuous data collection effort on food access, the risk and degree of food insecurity, and its adverse health effects among different refugee communities in the US.

## Data Availability

Data is not publicly available to protect participant privacy and ethical concerns.
